# Sexual Dimorphism in Brain Sirtuin-1 and m6A Methylated Sirtuin-1 mRNA, and in Protection with Post-Injury Anti-miR-200c treatment, after Experimental Stroke in Aged Mice

**DOI:** 10.14336/AD.2022.1225

**Published:** 2023-06-01

**Authors:** Lijun Xu, Xiaoyun Sun, Brian Griffiths, Ludmilla Voloboueva, Alex Valdes, Miles Dobrenski, Jeong-Jin Min, Creed M Stary

**Affiliations:** ^1^Department of Anesthesiology, Perioperative and Pain Medicine, Stanford University, School of Medicine, Stanford, CA 94305, USA.; ^2^Samsung Medical Center, Sungkyunkwan University School of Medicine, Seoul, Korea

**Keywords:** Sirt1, bioenergetics, neuroinflammation, miR-200c, sex differences, NAD^+^/NADH

## Abstract

We previously demonstrated that inhibition of miR-200c was protective against stroke in young adult male mice by augmenting sirtuin-1 (Sirt1). In the present study we assessed the role of miR-200c on injury, Sirt1, and bioenergetic and neuroinflammatory markers in aged male and female mice after experimental stroke. Mice were subjected to 1hr of transient middle cerebral artery occlusion (MCAO) and assessed for post-injury expression of miR-200c, Sirt1 protein and mRNA, N6-methyladenosine (m6A) methylated Sirt1 mRNA, ATP, cytochrome C oxidase activity, tumor necrosis factor alpha (TNFα), interleukin-6 (IL-6), infarct volume and motor function. MCAO induced a decrease in Sirt1 expression at 1d post-injury only in males. No differences in SIRT1 mRNA were observed between the sexes. Females had greater baseline miR-200c expression and a greater increase in miR-200c in response to stroke, while pre-MCAO levels of m6A SIRT1 was greater in females. Males had lower post-MCAO ATP levels and cytochrome C oxidase activity, and higher TNFα and IL-6. Post-injury intravenous treatment with anti-miR-200c reduced miR-200c expression in both sexes. In males, anti-miR-200c increased Sirt1 protein expression, reduced infarct volume, and improved neurological score. Conversely in females anti-miR-200c had no effect on Sirt1 levels and provided no protection against injury from MCAO. These results provide the first evidence of sexual dimorphism in the role of a microRNA in aged mice after experimental stroke and suggest sex-differences in epigenetic modulation of the transcriptome and downstream effects on miR biological activity may play a role in sexually dimorphic outcomes after stroke in aged brains.

## INTRODUCTION

Stroke remains the second leading cause of death worldwide, killing 6 million people and debilitating 17 million annually [1], and remains the primary cause of long-term disability in the US [2]. To date, the only pharmacological treatment for stroke approved remains tissue plasminogen activator, which must be administered within hours of stroke onset, and is associated with several contraindications. Although pre-clinical studies have identified hundreds of potential drug agents, no suitable alternative agents have been effectively translated into clinical therapy; there remains a critical need for novel drug alternatives that can overcome biological translational barriers. A major factor for translational failure is likely overwhelming use of male rodents in pre-clinical research and lack of sex-specific design and analysis in clinical trials. Stroke is an age-dependent, sexually dimorphic disease with evolving differences in incidence, prevalence, and outcome between males and females over the lifespan [3, 4]. Sex differences in stroke outcomes cannot be explained solely by circulating hormone levels, because the stroke incidence in women does not increase relative to men until decades after menopause. Furthermore, despite strong preclinical evidence of a protective role for estrogen, randomized clinical trials have failed to translate the beneficial effects of estrogen into therapy for stroke prevention in post-menopausal women. Moreover, treatment with estrogen led to an unexpected increase in stroke rates [5], therefore a dire need for novel therapeutic adjuvants in stroke prevention remains.

The molecular mechanisms underlying age-related sexual dimorphism in stroke are complex and remain undefined, but also offer an opportunity to leverage innate differences between sexes for novel drug development to improve outcomes in both sexes. MicroRNAs (miRs) are a class of non-coding RNAs that regulate post-transcriptional gene expression via translational silencing. We previously observed that miR-200c selectively increases in potentially salvageable peri-infarct regions after experimental stroke [6], and demonstrated that pre-injury miR-200c inhibition was protective against stroke in young-adult male mice [7]. Most recently we demonstrated that intravenous (IV) post-injury treatment with anti-miR-200c was also protective in young adult male mice after global cerebral ischemia,[8]. In the present study in order to extend the translational relevance of our prior observations to clinical stroke, we assessed the effect of post-stroke IV treatment with anti-miR-200c on protection against focal cerebral ischemia in clinically-relevant aged male and female mice.

MiR-200c is known to target the mitochondrial regulatory protein sirtuin-1 (Sirt1), a multifunctional NAD^+^-dependent deacetylase. Sirt1 functions as a bioenergetic sensor [9] and a regulator of mitochondrial function [10]. We previously demonstrated in young adult male mice that inhibition of miR-200c augmented astrocyte expression of Sirt1 after global cerebral ischemia and preserved downstream astrocyte mitochondrial function. Sirt1 has been shown to exert age-dependent effects on injury from experimental stroke in rats [11], however whether Sirt1 levels are associated with differential outcomes in stroke between sexes in the aged brain has never been assessed. Therefore, in the present study also we assessed acute and long-term cell-type specific Sirt1 expression in aged male and female mice after MCAO, and in response to miR-200c inhibition.

A primary mechanism of post-transcriptional modification and regulation of translational activity is methylation [12]. N6-methyladenosine (m6A) methylation is associated with alterations in miR activity [13] and prior studies have demonstrated that m6A modification regulates a variety of other RNA metabolic processes, such as mRNA stability [14], splicing [15], nuclear transport, and translational ability [16]. Sirt1 is a gatekeeper for m6A methylases [17] and changes in m6A methylation of mRNAs after experimental stroke have been described in young adult rodents [18]. In reverse, Sirt1 activity can also be determined by m6A methylation of its mRNA [19, 20], however whether sexual dimorphism exists in alteration of m6A methylation after experimental stroke in the aged brain has never been assessed. Therefore, in parallel we also measured m6A methylation of SIRT1 mRNA in aged males and females in response to experimental stroke.

## MATERIALS AND METHODS

### Transient Focal Cerebral Ischemia

All experimental protocols using animals were approved by the Stanford University Animal Care and Use Committee and conducted in accordance with the NIH guide for the care and use of laboratory animals. Aged (20-22-month-old) male or female CB57/B6 mice (Jackson Lab, Bar Harbor, ME) were randomized to treatment and anesthetized with 2% isoflurane in O_2_ by facemask. Focal cerebral ischemia was produced by 1h of transient middle cerebral artery occlusion (MCAO) using a silicone-coated 6-0 monofilament (Doccol, Sharon, MA) as described previously [21]. Cerebral blood flow (CBF) was monitored by non-invasive Doppler (Moor Instruments, Wilmington, DE). Any animals without evidence of decreased CBF were excluded from the study. Separate animals underwent sham surgery (anesthesia and incision but no MCAO). The rectal temperature was maintained at 37±0.5°C controlled by a homeothermic blanket control unit (Harvard Apparatus, Holliston, MA). Temperature and respiratory rate were monitored continuously. Mice with no evidence of acute neurological deficit or with evidence of hemorrhage were excluded from the study.

### Determination of Infarct Volume and Neurological Status

Cerebral infarction volume was determined at 1d post-MCAO with 2,3,5 triphenyltetrazolium chloride (TTC, Sigma, T8877, Louis, MO) after transcardiac perfusion with saline then fixation with 4% paraformaldehyde [22]. Infarct volume was quantified by a blinded observer assessing four 50μm coronal sections/brain and corrected for edema using Adobe Photoshop CS3 as described previously [23]. Neurological status was assessed after 1d of reperfusion by a blinded observer using a 4-point neurologic deficit score [22, 24, 25]. Neurological deficit was scored as follows: 0 - no observable neurological deficits, 1 - failure to extend right forepaw, 2 - circling to the right, 3 - falling to the right, 4 - cannot walk spontaneously.

### In vivo histological assessment

Animals were sacrificed at days 1, 3, 7 and 30 after MCAO by isoflurane overdose, and brains immediately perfused with transcardial ice-cold saline, then fixed with 4% phosphate-buffered paraformaldehyde (PFA) for stereological analysis. Coronal vibratome sections (50 μm) were used for fluorescent immunohistochemical (IHC) analysis as we have previously done [26]. Fixed sections were stained for the astrocyte marker glial fibrillary acidic protein (GFAP, #ab90601, Abcam, Boston, MA, 1:500 dilution), the mature neuronal marker NeuN (#ab104224, Abcam, 1:500 dilution) and Sirt1 (# ab189494, Abcam, 1:500 dilution) followed by secondary antibody solutions (Alexa Fluor 350nm, 488nm, and 594nm, Invitrogen, NY, USA, 1:500 dilution) in PBS at 4°C, overnight. Negative control sections received secondary antibody staining alone. Images were acquired by an observer blinded to conditions using an upright Zeiss Axio-Imager M2 fluorescent microscope equipped with Apotome 2.0 for optical sectioning, and Zeiss EC-Plan Neofluar 20X, Zeiss Plan Apochromat 40X, and Zeiss LC-Plan Neofluar 63X objectives. All imaging for a given protein were collected using a fixed excitation intensity, exposure time, and gain, to minimize variability. No post-imaging processing was performed. Qualitative Sirt1 protein expression co-localized with GFAP+ was performed using the “masking” function in Image-J v1.49b software (NIH, Bethesda, MD). In brief, Z-stack images were first independently analyzed in the GFAP channel, and then masked to generate GFAP+ regions of interest (ROIs). Next, GFAP+ ROIs were superimposed on the Sirt1 channel and then fluorescence intensities were measured and collated, for all sections. An observer blinded to conditions quantified from maximum projection Z-stack images the cell-type specific relative intensity of Sirt1 using StereoInvestigator™ (MicroBrightField, Williston, VT) software and Image-J v1.49b software (NIH, Bethesda, MD) as we have previously done [8].

### m^6^A methylation-RNA-immunoprecipitation (MeRIP) and RT-qPCR

Total RNA was isolated with TRIzol® (ThermoFisher Scientific, Waltham, MA) from ipsilateral brain tissue 1d after MCAO or sham surgery. Total m6A enrichment was performed using the Epigen™ Cut-and-Run kit (#P-9028) with 20mg total RNA and magnetic separation (DynaMag TM-PCR #49-202, ThermoFisher Scientific). Reverse transcription was performed as previously described [7] using the TaqMan MicroRNA Reverse Transcription Kit (Applied Biosystems, Foster City, CA). Predesigned primer/probes for PCR were obtained from ThermoFisher Scientific for mmu-miR-200c-3p (#4426961) and normalized to U6 small nuclear RNA (U6, #01973). Sirt1 (#4331182) was normalized to GAPDH (#4331182). PCR reactions were conducted as previously described [7] using the TaqMan® Assay Kit (Applied Biosystems). Measurements were calculated as the inverse log of the ΔΔCT from controls [27].

### Immunoblots

Total cellular protein was quantified by Pierce BCA protein assay kit (ThermoFisher Scientific). Equal amounts of protein were loaded and separated on 10-12.5% polyacrylamide gels, then transferred to Immobilon polyvinylidene fluoride membranes (EMD Millipore Corp, Burlington, MA). Membranes were blocked with 5% skimmed dry milk and incubated overnight with primary antibody against Sirt1 (Abcam, #ab110304) and β-actin (LI-COR Bioscience #926-42,210). Membranes were then washed and incubated with secondary antibodies (LI-COR Bioscience) followed by washing again and visualizing by using the LICOR Odyssey infrared imaging system. Densitometric analysis of bands was performed via Image Studio Lite (LI-COR Biosciences), and the intensity of all proteins was normalized to β-actin as a control.

### Measurement of Mitochondrial Function

Cytochrome c oxidase activity was determined using Cytochrome Oxidase Activity Colorimetric Assay Kit (BioVision, Milpitas, CA) according to the manufacturer’s instructions. Absorbance was measured at 550 nm every 30 sec for 300 sec. Cellular ATP concentrations were measured using the CellTiter-Glo luminescent ATP assay kit, based on the luciferase/luciferin reaction, from Promega (Madison, WI, USA) according to the manufacturer’s instructions. Luminescence was assessed using a multimode microplate reader (Spark 10M, Tecan Group LTD, Mannedorf, Switzerland).

### Neuroinflammatory Markers

Brains were removed and the ipsilateral hemisphere immediately homogenized in cold neuronal protein extraction reagent (ThermoFisher Scientific) using a ratio of 1g of tissue to 10ml of reagent. The samples were centrifuged at 10,000 × *g* for 20 min at 4°C and the supernatants were used for cytokine measurements. Levels of proinflammatory cytokines were determined by TNFα and IL-6 enzyme-linked immunosorbent assays (ThermoFisher Scientific).

### Statistical analyses

The number of animals per treatment group are identified in figure legends. Shapiro-Wilk and Kruskal Wallis tests were used to determine normality of data distribution. All data are reported as mean±SEM. Statistical difference was determined using T-test for comparison of two groups or 1-way analysis of variance (ANOVA) for multiple groups at one measurement time, or 2-way ANOVA with repeated measures for multiple groups with multiple time points. Analyses were followed by Bonferroni correction for experiments with N>2 groups using Sigmaplot (Systat Software, San Jose, CA, USA). P<0.05 was considered significant in all comparisons.

**Figure 1.**
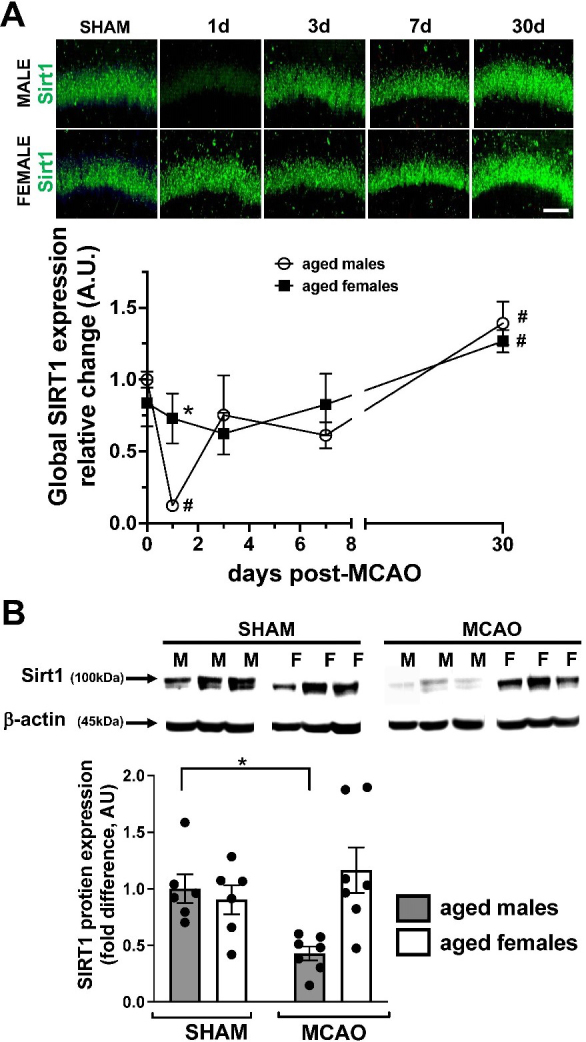
Sirtuin-1 (Sirt1) expression in aged male and female mice after middle cerebral artery occlusion (MCAO) in aged (20-22-month-old) mice. (A) Representative images (top) of hippocampal Sirt1 (green) and longitudinal measurements of Sirt1 expression in fixed brains using fluorescent immunohistochemistry (bottom). N=3-4 animals per group, each data point represents averaged results from 5 separate sample images per animal, mean+SEM, *p<0.05 versus male within given time point, #p<0.05 versus sham within sex, 2-way analysis of variance (ANOVA) ANOVA with repeated measure. (B) Measurements and examples of Sirt1 protein expression 1d after MCAO using immunoblot with b-actin as internal control. N=6-7 animals per group. Mean+SEM, *p<0.05, 1-way ANOVA. Scale bar = 50 µm.

**Figure 2.**
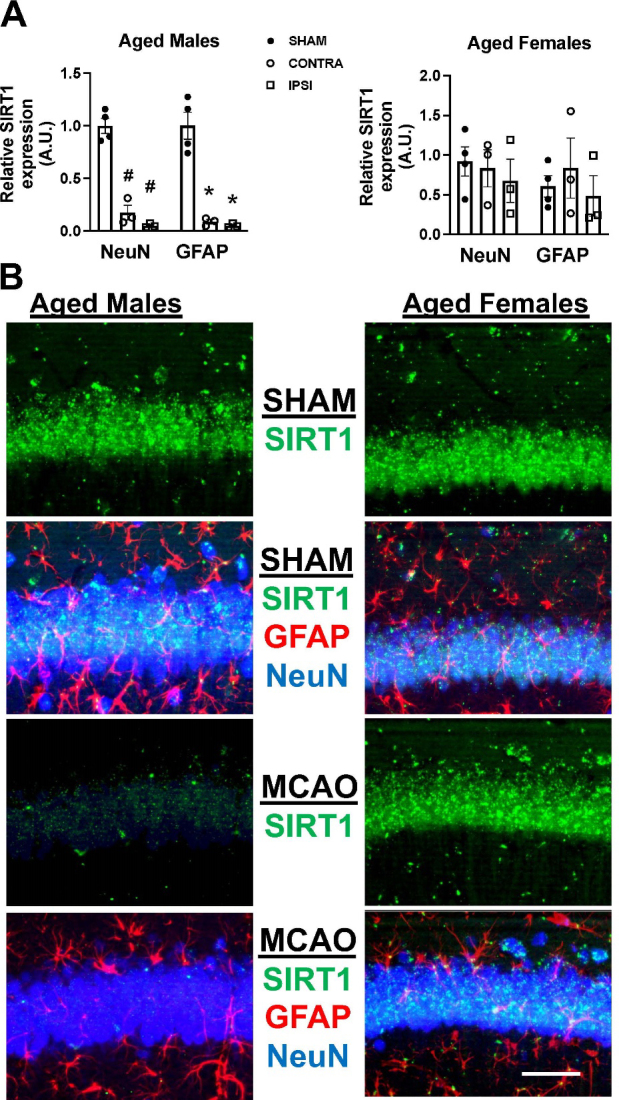
Cell-type and regional-specific Sirt1 expression after MCAO in aged male and female mice. (A) Levels of Sirt1 expression co-localized with neurons (NeuN+) and astrocytes (glial acidic fibrillary protein, GFAP+) in ipsilateral (MCAO ipsi) and contralateral (MCAO contra) brain 1d after MCAO in aged males (left) and females (right). (B) Representative IHC images of hippocampal cornu ammonis 1 stained for Sirt1 (green), NeuN (blue) and GFAP (red) in aged males (left panels) and females (right panels) 1d after sham surgery (top four panels) or MCAO (bottom four panels). N=3-4 animals per treatment group, each data point represents averaged results from 5 separate sample images per animal, mean+SEM, *p<0.05 versus sham, 1-way ANOVA. Scale bar = 25 µm.

## RESULTS

### Sexual dimorphism in acute post-injury expression of Sirt1 in the aged brain after MCAO

Sirt1 expression in the brain was assessed in aged male and female mice at 1d, 3d, 7d, and 30d after MCAO by IHC (Fig. 1A). No baseline differences in Sirt1 expression were observed between male and female sham animals, however Sirt1 expression was significantly (p<0.05, 2-way ANOVA) reduced at 1d after MCAO only in males. Conversely Sirt1 levels in both males and females were significantly elevated at 30d compared to sham animals. Sexual dimorphism in Sirt1 levels at 1d post-MCAO was confirmed quantitatively by immunoblot (Fig. 1B).

**Figure 3.**
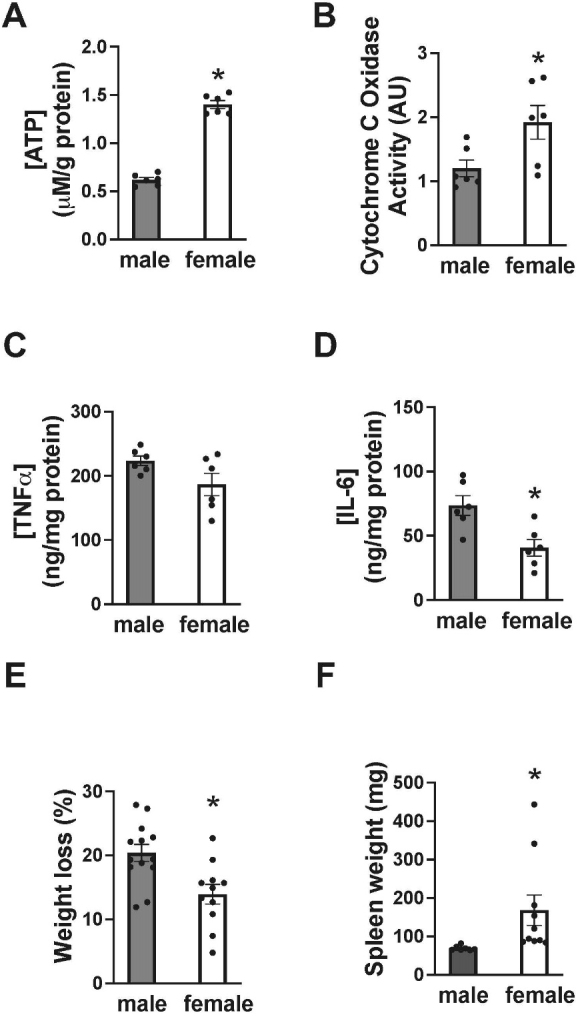
Comparisons of bioenergetic, neuroinflammatory and physiologic responses to MCAO in aged male and female mice. Brain ATP levels (A), cytochrome C oxidase activity (B), tumor necrosis factor alpha (TNFα) levels (C), and interleukin-6 (IL-6) levels (D) 1d after MCAO in aged male and female mice. Percentage weight loss (E) and spleen size (F) 3d after MCAO in aged male and female mice. N=6 animals per group, mean+SEM, *p<0.05, Students t-test.

### Sex-differences in post-MCAO Sirt1 expression occurs across cell-types and regions

Next, we assessed regional (ipsilateral and contralateral hippocampal) Sirt1 co-localization with NeuN+ neurons and GFAP+ astrocytes in aged males and females at 1d after MCAO with IHC (Figs. 2A, B). Compared to sham, we observed a significant (p<0.05, ANOVA) decrease in Sirt1 in both NeuN+ and GFAP+ cells, in both hemispheres, only in males. Conversely no significant differences were observed in regional or cell-type specific Sirt1 expression in females at 1d post-MCAO compared to sham females.

### Bioenergetic, neuroinflammatory and physiologic parameters suggest greater injury in aged males compared to aged females

We next compared ipsilateral brain levels of ATP (Fig. 3A), cytochrome C oxidase activity (Fig. 3B), and levels of the pro-inflammatory cytokines TNFα (Fig. 3C) and IL-6 (Fig. 3D) between aged males and females 1d after MCAO. We observed significantly (p<0.05, Students t-test) lower post-MCAO ATP and cytochrome C oxidase in males compared with females, suggesting greater impairment in bioenergetic capacity. Conversely, we observed significantly (p<0.05, Students t-test) greater levels of post-MCAO TNFα and IL-6 in males relative to females, suggesting a greater degree of neuroinflammation. Also underscoring greater injury in males, post-MCAO weight loss was significantly (p<05, Students t-test) greater (Fig. 3E) and spleen size significantly (p<0,05, Students t-test) smaller (Fig. 3F) in aged males versus aged females.

**Figure 4.**
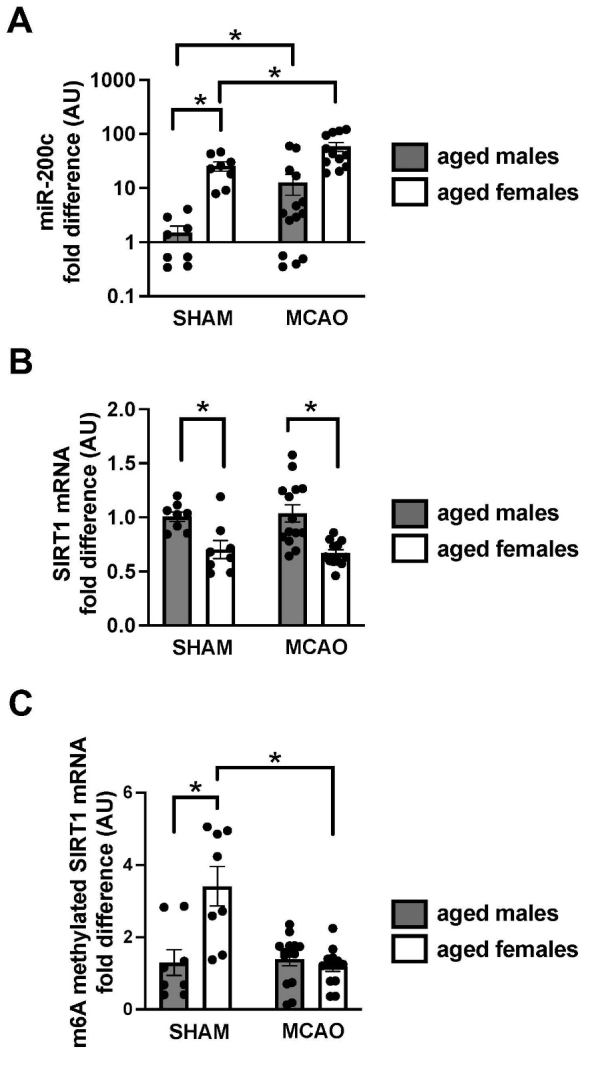
Post-MCAO expression of miR-200c, SIRT1 mRNA and m6A methylated SIRT1 mRNA in aged male and female mice. Fold difference in miR-200c expression (A), SIRT1 mRNA levels (B), and m6A methylated SIRT1 mRNA levels (C) in aged males and females after MCAO (relative to sham males). N=7-14 animals per treatment group, mean+SEM, *p<0.05 Students t-test.

**Figure 5.**
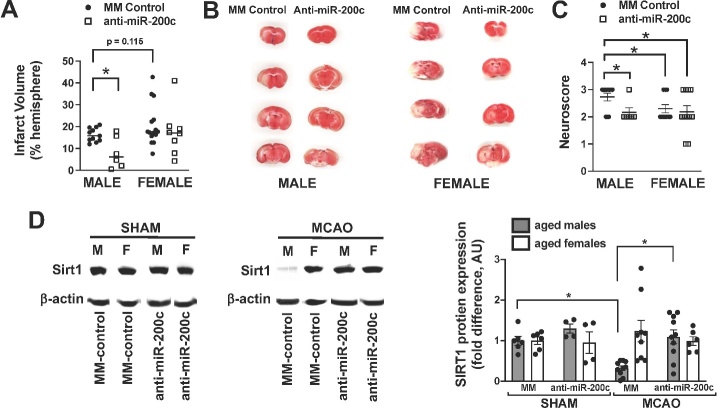
Effect of 2h post-MCAO treatment with intravenous anti-miR-200c on injury and Sirt1 expression in aged males and females. (A) Infarct volume in aged males and females 1d after MCAO and 2 h intravenous post-treatment with either anti-miR-200c or mismatch control sequence (MM control). N=6-14 animals per treatment group, mean+SEM, *p<0.05, Students t-test for A and C. Examples of coronal brain sections stained with the metabolically active indicator 2,3,5 triphenyltetrazolium chloride (TTC, red, (B) and neurological scores (C) in aged males and females 1d after MCAO and post-injury treatment with anti-miR-200c or MM control. (D) Examples from Sirt1/β-actin immunoblots (left) and quantification of Sirt1 proteins levels (right) from ipsilateral brains of aged males and females 1d after MCAO and post-injury treatment with intravenous anti-miR-200c or MM control. N=4-10 animals per treatment group, mean+SEM, *p<0.05, 1-way ANOVA for D.

### Epigenetic differences in the response to MCAO between aged males and females

In order to identify the underlying molecular mechanisms that could regulate sexual dimorphism in Sirt1 and bioenergetics after MCAO we next compared brain levels of miR-200c (Fig. 4A) and SIRT1 mRNA (Fig. 4B). We observed significantly (p<0.05, Students t-test) greater baseline levels of miR-200c in sham aged female brains compared with sham aged male brains, with subsequent significantly (p<0.05, Students t-test) greater post-injury miR-200c increases in females relative to males (Fig. 4A) at 1d after MCAO, despite unchanged post-MCAO Sirt1 protein levels in females (Figs. 1 and 2). In contrast, SIRT1 mRNA levels were significantly (p<0.05, Students t-test) lower in both sham females and post-MCAO females versus treatment-matched aged males (Fig. 4B). Notably SIRT1 mRNA levels remained unchanged after MCAO in both groups, also in contrast to our observations of decreased Sirt1 protein expression in males (Figs. 1 and 2). To further investigate these observations, we compared levels of m6A methylated SIRT1 mRNA (Fig. 4C), as differences in translational efficiency have been attributed to differences in m6A methylation [12]. We observed significantly (p<0.05, Students t-test) greater levels of baseline m6A methylated SIRT1 mRNA in sham aged females versus males (Fig. 4C). This difference normalized by a significant (p<0.05, Students t-test) post-MCAO decrease in methylated SIRT1 mRNA only in females, while post-MCAO levels in males remained unchanged compared to sham.

### Post-MCAO IV treatment with anti-miR-200c preserves Sirt1 and protects against injury only in aged male mice

Post-injury IV treatment significantly (p<0.05, Students t-test) reduced brain miR-200c levels 1d after MCAO in both males (1.9±0.3-fold change from sham versus 8.10±3.1-fold change for MM-control) and females (1.07±0.2-fold change from sham versus 50.39±13.3A for MM-control). A 1hr period of MCAO resulted in significant infarct volumes in both males and females, with no statistical difference between groups (Figs. 5A, B). Post-injury IV treatment with anti-miR-200c reduced infarct volume in males but did not alter infarct volume in females (Fig. 5C). Males also had significantly improved neurological scores after MCAO with treatment by anti-miR-200c (Fig. 5B) while females had no significant difference in neurological scores compared with MM-control treatment. Anti-miR-200 treatment was also associated with augmented post-MCAO Sirt1 expression in males relative to MM-control treated aged males (Fig. 5D), while no differences were observed in brain Sirt1 levels with anti-miR-200c treatment in aged females.

## DISCUSSION

Despite growing epidemiologic and clinical evidence demonstrating sexual dimorphism in the incidence and response to stroke, experimental studies in rodents have traditionally employed only young male animals, due in part to high animal costs and difficulty performing surgeries in aging animals [28]. However, the exclusion of clinically relevant aged and female populations has likely contributed to the translational barriers in the development of novel clinical stroke therapeutics. Although limited, prior studies in aged animals have recapitulated age-dependent clinical sexual dimorphism in stroke outcomes [28, 29]. While young adult female mice have estrogen-dependent smaller infarct than males [22, 30, 31], aged male and female mice have generally equivalent infarct volumes after MCAO [32]. In the present study we observed evidence of greater neuroinflammation in aged male brains with elevated IL-6 and TNFα 1d after MCAO versus aged female brains. This is in contrast with observations by Liu *et al*., who observed greater injury and IL-6 in aged female mice [28]. These contradictory findings may be due to differences in age (16-month-old versus 20-22-month-old in the present study), duration of MCAO (90 min versus 60 min), and/or in suture size used for MCAO (5-0 versus 6-0 monofilament, respectively) between their prior study and ours. Previous observations in mice have indicated that the percentage body weight loss post-MCAO correlates with stroke lesion volume [33]. Our observations in the present study of greater weight loss in males versus females supports our conclusion of greater injury in the 20-22-month-old males used in the present study. These observations underscore the crucial importance of methodological consistency and age-matching in experimental stroke applications in aged pre-clinical stroke models.

Supporting our observations of greater neuroinflammation after MCAO in aged males, in the present study we also observed lower levels of ATP and decreased cytochrome C oxidase activity in males, indicating greater impairment in bioenergetic state in males versus females. Mitochondria are the site of ATP synthesis, regulate oxidative stress and play a key role in cell death after stroke brain injury [34-36]. Young adult female brain tissue has higher baseline mitochondrial respiration and lower oxidant production compared to young adult male brain, however these differences become suppressed with age [37, 38]. Prior work [39, 40] in young adult rodents has demonstrated that cell death signaling pathways following cerebral ischemia are sexually dimorphic [39, 40] however comparative studies of post-MCAO brain mitochondrial function in aged animals are lacking. These previous observations and the findings from the present study strongly suggest that future comparative studies elucidating sex differences in post-stroke mitochondrial function in aged brains could be a promising avenue for discovery of novel sex-specific and sex-independent approaches to mitigate injury after stroke.

As a bioenergetic sensor and gatekeeper of mitochondrial dynamics, Sirt1 plays a central role in maintaining mitochondrial and cellular homeostasis after ischemic injury. Pre-clinical studies in young adult male animals [41-43] support the hypothesis that activation of Sirt1 can be protective against stroke, however more recent evidence indicates any protective effect is both age- [44] and sex-dependent [11]. In the present study we observed significant decreases in Sirt1 1d after MCAO only in aged male brains, while post-injury Sirt1 levels in female brains remained stable. This observation could, at least in part, explain prior observations of differential protection against MCAO with Sirt1 activation between ages and sexes [11, 44]. Mechanistically, activation of Sirt1 could result in competing downstream effects on mitochondrial homeostasis: for example Sirt-1 mediated activation of AMP-dependent kinase serves to balance ATP demand with ATP availability, while conversely Sirt1 depletes NAD^+^ availability via augmented NAD^+^-dependent de-acetylase activity [45], which in turn reduces oxidative phosphorylation capacity. Interestingly we observed significantly elevated Sirt1 in both males and females 30d after MCAO, which may indicate a restorative process, as Sirt1 is also central to post-stroke metabolic regulation and mitochondrial biogenesis by activating peroxisome proliferator-activated receptor gamma coactivator 1-alpha [46]. Alternatively, our observation of elevated Sirt1 levels in both sexes could be simply due to survivor bias. Further comparative mechanistic and long-term studies in post-stroke aged male and female brains investigating the effects of Sirt1 overexpression, activation, and inactivation are warranted.

While miR-based studies in aged models are increasing, comparative studies between aged males and females remain limited [47]. We and others previously demonstrated in young adult mice that expression of Sirt1 translation is inhibited by miR-200c [8]. Levels of miR-200c are strongly influenced by oxidative stress [6, 7, 48], and in the present study we observed significant increases in miR-200c in brains of both aged males and females after MCAO. We previously demonstrated that both pre-injury intracerebroventricular treatment [7] and post-injury IV treatment with anti-miR-200c was protective against cerebral ischemia in young adult male mice [8]. In the present study we have identified, for the first time, sexual dimorphism in efficacy with miR inhibition in protection against stroke in clinically relevant aged animals. MiR-200c is a central regulator of mitochondrial function, at least in part via upstream regulation of Sirt1 [8]. To support this observation, we observed augmented Sirt1 in males with anti-miR-200c treatment in parallel with protection. However, paradoxically, we observed lower levels of SIRT1 mRNA expression despite higher Sirt1 protein expression in females. One mechanism to explain the differential effects of anti-miR-200c on protection and Sirt1 expression could be sexual dimorphism in translational suppression of Sirt1 by miR-200c.

Post-transcriptional modification of mRNAs is a central mechanism for refinement of mRNA translation via modulation of miR binding affinity for the mRNA 3’ untranslated region. A primary mechanism of post-transcriptional modification and regulation of translational activity is m6A methylation [12]. A recent study in young adult mice demonstrated sexual dimorphism in m6A control of metabolism [49] while a separate recent study demonstrated increased m6A sites with aging, in both mouse and human [50]. Comparative studies of m6A methylation in aged brains in response to MCAO are lacking, however in the present study we observed for the first time in aged mice sexual dimorphism in m6A methylated SIRT1 mRNA levels in response to MCAO. Whether this effect relates to our observations of the differential response of Sirt1 to MCAO between sexes, and/or for the sexually dimorphic effect of anti-miR-200c in protection against MCAO is unknown but does provide directionality for future investigations. To support this, in young adult male mice, Chokhalla *et al.* [18] observed significant increases in global m6A levels and transcriptome-wide m6A hyper- and hypomethylation changes after MCAO. Whether differences in m6A methylation of other mRNAs, or other epigenetic alterations in mRNA known to regulate RNA binding affinity and translational efficiency, including N4-acetylcytidine RNA methylation [51] and N4-acetylcytidine RNA acetylation [52], could account for our observations of differential Sirt1 expression and/or protection by anti-miR-200c between aged males and females are unknown. Future studies addressing this novel intersection between miR biology and epigenomic changes in the context of sexual dimorphism in mitochondrial dysfunction and injury after stroke could provide several keys to unlocking novel, effective treatments for both men and women survivors of stroke.
